# Counselling in a glaucoma care service

**Published:** 2022-01-31

**Authors:** Fatima Kyari, Heiko Philippin, Peter Shah, Hannah Faal, Sani Babayo, Mohammed Abdull

**Affiliations:** 1 Associate Professor: International Centre for Eye Health, London School of Hygiene & Tropical Medicine, UK. Consultant Ophthalmologist: College of Health Sciences, University of Abuja, Nigeria.; 2 Clinical Research Fellow: International Centre for Eye Health, London School of Hygiene & Tropical Medicine, UK. Global Advisor for Inclusive Eye Health/Research & Training: CBM, Bensheim, Germany and Glaucoma Specialist: Eye Center, Medical Center, University of Freiburg, Germany.; 3 Consultant Ophthalmic Surgeon: University Hospitals Birmingham (UHB) NHS Foundation Trust, Birmingham, UK and President of the UK and Eire Glaucoma Society.; 4 Professor of International Eye Health: University of Calabar, Calabar, Nigeria.; 5 Senior Social Welfare Assistant Officer: National Eye Care Centre, Kaduna, Nigeria.; 6 Chief Consultant/Associate Professor: Ophthalmology Department, Abubakar Tafawa Balewa University, Bauchi, Nigeria.

**Counselling can improve the eye health and quality of life of patients with glaucoma.**

**Figure:**
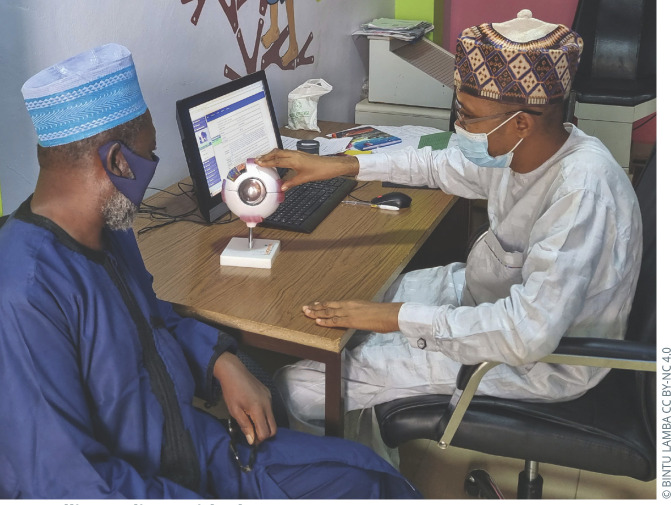
Counselling a client with glaucoma. **NIGERIA**

Glaucoma patients often think that their condition is synonymous with blindness and disability, leaving them feeling worried and vulnerable. They may also develop mental health issues such as depression or anxiety. Unfortunately glaucoma patients may have mental health issues even if these concerns are addressed. The glaucoma care team must offer patients balanced information that will help them to understand their options, regain hope for their future, and take practical action to protect their eyes and vision. This support – known as counselling – will help to improve patient’s quality of life.

## Who should counsel glaucoma patients?

In a typical, busy clinic, some aspects of counselling can be delivered by examining clinicians, supported by other members of the glaucoma care team. However, it is preferable to give patients access to a dedicated counsellor (who may also offer counselling about other diseases and postoperative care).

Ideally, the glaucoma care team should train someone to take on this role. The person must be approachable, be skilled at talking to patients and their families, and must understand glaucoma and its treatment. Nurses, social welfare, or community health workers could be good candidates for training as glaucoma counsellors.

It is vital that clinical personnel on the glaucoma care team are in close contact with the person responsible for counselling and that they share any relevant clinical information (about the patient) with the counsellor. This enables the counsellor to answer any questions the patients may have, and/or to ask the clinicians for more information if anything is unclear.

## The purpose of counselling

Counselling can be helpful in several ways, including:

- To help patients understand their condition and accept their prognosis; this may sometimes include coming to accept that the vision they have lost cannot be restored.
- To find out what patients need or want to do and refer them to other services that may help (e.g. low vision or rehabilitation).
- To provide information and help them make a decision about treatment, such as surgery.
- To improve patients’ compliance with their medical treatment (e.g. regularly instilling eye drops in the correct way).

## Focusing on the patient and their needs

A list of information can be helpful and complimentary to counselling and is not necessarily the opposite of counselling. Counselling is, by its nature, patient-centred: the counsellor is focused on the patient, what they know and understand, what they want to do, and what they need in order to have good eye health and to live as well as they possibly can.

Patients’ family members and carers should always be included in counselling and it is important to listen to them. Ask them questions and find out how they intend to help the patient to achieve her or his goals. Another reason for including family members is that suffering from vision impairment or blindness without a visible cause such as a white pupil (as in cataract) can lead to alternative explanations, e.g. laziness or curses, and this can result in the patient being stigmatised by others. Red eyes from eye drops can be mistaken for an alcohol problem, for example. Constant and expensive treatment and visits to the glaucoma care team, seemingly without improvement, may be difficult for the patient to explain.

It may be necessary to provide the same information several times, particularly shortly after diagnosis, when an asymptomatic patient may be in denial. We have to be careful, however, as providing the same information, in the same way, may not have the desired effect – patients may stop listening if the information seems over-familiar. Finding out how patients have used the information in their lives, or how it applies to them, makes for a better approach (see Table 1).

**Table 1:** Tips for explaining glaucoma to patients: what to ask them, what to tell them (depending on what they said), and why it matters.

What to ask patients	What to tell them (adapt this depending on what they have told you)	Why this is important
**Can you tell me what you know about glaucoma?**	Glaucoma typically develops because of increased eye pressure, often because of a reduced outflow from the eye. Reducing the eye pressure can slow down the progression.	When a patient is diagnosed with glaucoma, it often has a negative impact on their quality of life. It is important to explain the causes, how it progresses, and the patient’s individual prognosis so that they are not unnecessarily anxious but will also take any treatment seriously.
**What symptoms made you come to the hospital? Do you know other ways that glaucoma patients can present?**	Glaucoma starts very gradually but will get worse over time – it is a progressive disease. In the early stages there may be no symptoms until there is more damage. Vision impairment, which occurs late in the disease, may be the first symptom that brings most people to hospital, but some can recognise a reduction (constriction) in their visual field and come early. Other symptoms may include dullness, aches and pain in the eyes, problems with colour vision, proneness to accidents (from stumbling on objects when walking), haloes (rainbow colours) around point light sources, etc.	Explaining to patients what the symptoms are may help them to educate others, so that people with similar symptoms may be more more likely to report to hospital early. It can also help patients to talk to their relatives and friends about their specific needs, for example the need to remove obstacles at home.
**What do you know about the differences between cataract and glaucoma?**	Surgery for cataract (‘white’ blindness) involves replacing the opaque lens (often visible to the patient) with a clear lens, and vision is restored. With glaucoma (‘black blindness’), treatment (including surgery) stops the vision from getting worse. Vision that is lost cannot come back.	Glaucoma and cataract are often confused and facts about cataract might be more widely known in some regions. This article and a poster from a previous edition of this journal, can be used to explain the difference to patients. **www.cehjournal.org/article/what-is-wrong-with-my-vision-and-what-can-i-do/**
**What, in your opinion is the aim of treatment for glaucoma?**	Treatment of glaucoma does not improve vision and sometimes progression can only be slowed down. But with regular visits and treatment, vision can often be preserved. Follow-up and treatment must be pursued life-long.	Many patients expect an improvement of their vision after treatment. Without explanations, they might assume that the condition is treated with a single bottle of eye drops or may stop if a few months of treatment has not improved their sight.
**Is someone else in your family having a similar problem?**	Glaucoma may be more common in some families because the disease may be inherited. However, this does not mean that everyone will inevitably have it. This is why it is important for you to advise your first-degree relatives to find time and come to the hospital to check if they have glaucoma. If they come early on, we can treat them before they lose any of their sight.	A positive family history might help the patient to accept the diagnosis and family members can motivate each other to seek counselling and care. Often, a positive family history will help in reducing stigma associated with the disease as other family members have a better understanding of the problem. It also helps the family as a group to reinforce the individual’s management of the disease. It is important to ensure that patients and family members understand what can be done to prevent glaucoma from resulting in vision impairment.
**Do you sometimes see patterns, objects, or people that you know do not exist, or which other people don’t see?**	This is known as Charles-Bonnet syndrome, and people who have very advanced glaucoma can be affected. You are seeing these images because of the damage to the nerve at the back of your eyes. You may notice that the images are smaller than you would normally expect; this is typical of the syndrome, and it really is nothing to be concerned about. If you want, I will explain this to your carers and loved ones.	Symptoms of Charles-Bonnet syndrome are caused by cortical stimulation without visual input which leads to visual hallucinations (not auditory, nor olfactory) in people with advanced and end-stage glaucoma. Patients understand that the images are not real and often do not report them because they fear mental illness and possible stigmatisation. The symptoms can also be misinterpreted by those around the patients.

Where possible, avoid giving information that sounds like a command. For example, instead of telling a patient to “use your medication or you will go blind”, ask them to tell you what they know about the consequences of not adhering to treatment. Listen carefully, then follow up by asking what they think they can do to avoid these consequences. After you have listened to the patient, reflect back to them what they have said (“So, what I heard you say is that …”). Then you can explain further or correct any misunderstanding.

## Explaining the disease

Take care not to overload patients with facts – only give information that they can understand and apply in practice. Start by asking patients what ideas they have about glaucoma and about the subject of the counselling session (e.g., their own prognosis, acceptance of a surgical procedure, or compliance with treatment), and go on from there.

Glaucoma also has a genetic component, and it is important to discuss this carefully and with great sensitivity. Knowing that glaucoma can occur in many family members is helpful as patients can encourage their relatives to come for screening. However, if glaucoma is seen as a condition that will inevitably lead to blindness and disability, there is a risk of stigma which can lead to family difficulties. For example, it could cause problems in a relationship if there are fears about passing the condition on to children. Therefore, the counsellor must be tactful and guide the discussion towards the identification of first-degree relatives, in particular.

**Top tips**

- Develop a rapport with the person. There should be a relationship of trust – a therapeutic relationship. Listening is vital. Allow the patient to express themselves in their own words. This is also a way of finding out how they understand the situation, and where there may be gaps in their knowledge.
- Explain the disease to them in terms that are easy to understand (see From the Field panel for an example).
- Listen to the patient. Try to understand how they see their disease and its treatment, as well as their interaction with the health system.
- Try and determine what their ‘soft spots’ are – what matters to them? For example, their children, their job, or looking after an older relative? Explaining how adhering to treatment would benefit them and the people or things they care about, may be helpful motivation for them to take action or change their behaviour.
- You may have given them some bad news about vision that is already lost, and which may be lost in future. You must give them time and space to go through the various stages of grief: denial, anger, bargaining, depression and then acceptance. Only when they accept this can they take action.

## Encouraging compliance with medical treatment

When discussing eye medication, it is important to ask the patient how often they use their medication and to invite them to demonstrate how they instil it. You can then show them the correct way to do it.

Patients’ ability to instil medication correctly may be affected by several factors, e.g., arthritis of the hands, and it is important to suggest adaptations that can help them, or to train family members to do it for them. Family members can also remind the patient when it’s time to instil their eye medication, which will help to improve their adherence to medication regimes and improve the effectiveness of their treatment.

The PDF of this article (**www.cehjournal.org/article/instilling-your-own-eye-drops**) can be printed and given to patients as a guide.

## Low vision and rehabilitation

If the patient has vision impairment, refer them for low vision services – low vision services can provide equipment and training to help them make the best use of the vision available to them. Patients may also benefit from rehabilitation services, where available, and can learn skills such as reading Braille or using a keyboard, typewriter, or other adaptive technologies.

## Daily living

A counsellor can also support the patient to adapt to their condition and improve their circumstances. Instead of listing the different environmental modifications they can make, it is better to find about their present condition and how they can adapt, depending on their needs. What do they struggle with, and what do they want to be able to do? For example, finding their way from their home to a friend’s house, or taking care of their physical appearance (personal grooming).

This discussion should be about concrete ideas that the patient can carry out with what they have available. You can make suggestions, but it is also important to elicit from the patient and their family members or carers what they can to achieve that change.

Patients may benefit from joining **support groups for people with glaucoma**. These are often organised by patients for patients and their relatives, and sometimes they are facilitated by eye care providers such as nurses. Here patients can learn more and share their own experience with others. They can have a positive impact on someone by, for instance, sharing techniques used in maintaining adherence to medication, procurement of medication, or adapting their environment and lifestyle. Find out what groups are available in your area and encourage patients to join them.

Peer mentoring is another useful strategy. This is where the patient is connected to another person with a similar condition to provide guidance towards developing self-help or personal grooming skills, for example.

**From the field**

**How I explain glaucoma to clients**

**Figure:**
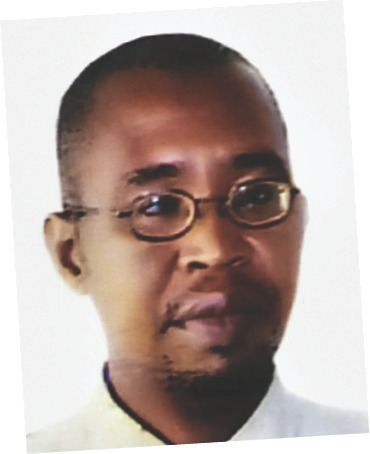


**Sani Babayo**

Senior Social Welfare Assistant Officer: National Eye Care Centre, Kaduna, Nigeria.

Glaucoma patients are referred to me for counselling by the ophthalmologist. This means that before a patient is referred for counselling, they would have been seen and diagnosed by the ophthalmologist and started on treatment. I call them ‘clients.’ I first of all establish rapport by listening to them and determining where their main concern is. I then correlate with the information provided to me by the ophthalmologist.

I start by explaining the eye and intraocular pressure (IOP), vision, and the role of the optic nerve. The optic nerve is within the visual pathway and acts like the wire for a video player. The visual inputs are formed in the brain.

Specifically for glaucoma, I go on to explain the trabecular meshwork and the drainage system for aqueous draining. The analogy is like the gutter system in one’s house – if it is clogged, there is a build-up of water (aqueous). The increase in the amount of water leads to an increase in pressure. In the case of the IOP, it has an effect on the optic nerve. Imagine the optic nerve in rings/layers as an onion. Not all the layers are affected at the same time; the damage starts with the inner layers thereby disrupting the transmission of visual input to the brain. The higher the IOP, the more the layers affected, and the greater the vision loss. This helps the client to understand how the loss of vision happens and where the damage occurs.

**“I allow the client to express whether they understand. I watch their expression, ranging from anger to denial and acceptance, depending on their expectations.”**

I explain that optic nerve damage does not recover. I do not tell them directly that their lost vision will not be recovered. I also try to relate their visual acuity and/or visual field with the level of their cup:disc ratio (indicated by the ophthalmologist). Next, I allow the client to express whether they understand. I watch their expression, ranging from anger to denial and acceptance, depending on their expectations. I note their gestures more than just hearing their words.

I then explain, in detail, their treatment as indicated by the ophthalmologist – the aim of treatment is to maintain the present vision. If they are severely vision-impaired or blind, I talk them through activities of daily living.

Sometimes, I make a self-disclosure i.e. by sharing my own story – of what is relevant to them.

The feedback from my patients has been very positive. For example: “Now I understand the disease so I know the relationship between the eyedrops and the rest of my life. I am able to correlate the worsening disease with [problems in] the use of my medication.”

**From the field**

**My experience counselling patients using adapted motivational interviewing**

**Figure:**
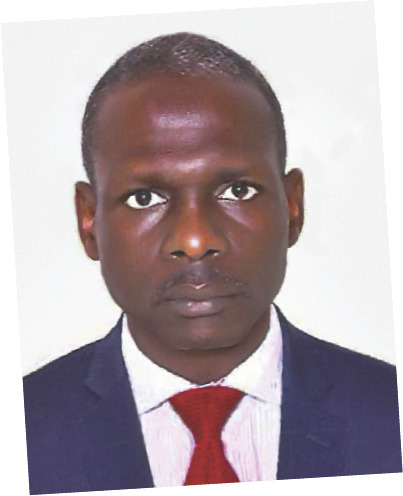


**Mohammed Abdull**

Chief Consultant/Associate Professor: Ophthalmology Department, Abubakar Tafawa Balewa University, Bauchi, Nigeria.

As a clinician dealing with glaucoma patients, I never had any real formal training in counselling or how to talk to patients. The training most doctors have is to give directives or information to patients, not really knowing whether the patients grasp what we are communicating or not. When patients do not return for follow up, or fail to use their medication as prescribed, we tend to show concern for their health or sometimes even get angry with them for not abiding by our instructions. We get surprised when, despite the severity of the patient’s problems, they fail to adhere to medication or instructions. We talk to patients, give them materials to read but all to no avail. We have the tendency to give too much and often unnecessary information to patients about glaucoma, even when they do not ask for it.

I discovered motivational interviewing during my academic training. Motivational interviewing is an approach to counselling that helps people to make positive changes in their own behaviour to support their health and wellbeing. It involves:

- **Engaging** with the patient and creating a partnership or alliance between the counsellor and the patient.
- **Focusing** on what needs to change – what is the most important target for change? This could be taking medication more regularly, or practicing the correct way to instil eyedrops.
- **Evoking motivation** – working with the patient to find what motivates them to change. Why do they want to make this change? What will be the impact on the things and people that matter to them?
- **Planning** – helping the patient to make concrete plans to make the change and maintain it in future.

Another issue is that many of our patients are used to being directed to do things, so they find it strange that I should ask their view on something or what they intend to do. In many patients there were clear signs of discomfort, as they thought they were being assessed. Their expectation is to be told what to do by us, not the other way round. An elderly woman in the clinic once said to me “Please doctor, stop asking me what I would do. Just tell me what I should do”. Some patients will open up and talk about the eye problem, how it started or presented, what they did, etc., but not what they would do to help themselves. Some don’t want to disappoint you, so they say what they think we want to hear; it becomes a challenge to get them to say what is really going on. They may think that, as their doctor, I will not be happy if they told the truth that they have not been using their medication regularly.

**“Many of our patients are used to being directed to do things, so they find it strange that I should ask their view on something or what they intend to do.”**

Some patients depend on family members, guardians, or husbands to the extent that they cannot show intent to commit to anything on their own. They lack a sense of agency (feeling able to act on their own behalf) and are not used to having their opinions heard or being involved in making decisions, even about their own health or welfare. With empathy and patience of a trained counsellor, such patients tend to do very well. Furthermore, to avoid “white coat fear”, counselling is better carried out by trained counsellors instead of doctors.

Counselling methods that give positive results should be enhanced and perfected for continued use. The ultimate aim is the welfare of our patients, prevention of blindness, and improvement in their quality of life.

*The author and his colleagues carried out a study^1^ to assess the impact of adapted motivational interviewing on acceptance of surgery, adherence to treatment, coming for follow up and ultimately control of intraocular pressure in glaucoma patients.*

Reference1AbdullM. M., et al. (2017). “Can Adapted Motivational Interviewing Improve Uptake of Surgical or Laser Treatment for Glaucoma in Nigeria: Randomized Controlled Trial.”
Journal of Glaucoma
26(9): 822–828.2885794510.1097/IJG.0000000000000729
